# Dying the right-way? Interest in and perceived persuasiveness of parochial extremist propaganda increases after mortality salience

**DOI:** 10.3389/fpsyg.2015.01222

**Published:** 2015-08-14

**Authors:** Lena Frischlich, Diana Rieger, Maia Hein, Gary Bente

**Affiliations:** ^1^Department of Psychology, University of CologneCologne, Germany; ^2^University of MannheimMannheim, Germany; ^3^Academia for Applied Psychology and Psychotherapy, CologneGermany; ^4^Michigan State University, East Lansing, MIUSA

**Keywords:** Parochial altruism, mortality salience, right-wing extremism, propaganda, persuasion

## Abstract

Research on parochial altruism demonstrated that hostility toward out-groups (*parochialism*) represents the dark side of the willingness to benefit one’s in-group even at own costs (*altruism*). Parochial aggression thereby emerged mainly under conditions of threat. Extremist propaganda videos, for instance by right-wing extremists, try to capitalize on parochial altruistic mechanism by telling recipients sharing their national identity that this nation is under threat wherefore they for have to join the extremist’s cause to prevent the extinction of their nation. Most of the time, propaganda videos are rated as uninteresting and non-persuasive by the target audience. Yet, evolutionary media psychology posits that the interest in and effectiveness of media increases when evolutionarily relevant problems are addressed. Consequently, interest in parochial altruistic right-wing extremist messages should increase under conditions of threat. The current study tested this assumption by randomly assigning German non-Muslims (*N* = 109) to either an existential threat (here: mortality salience) or a control condition and asking them to evaluate extremist propaganda that addressed them as either in-group members (right-wing extremists) or as out-group members (Islamic extremists). In support of the hypotheses, subjects under conditions of threat reported a higher interest in the right-wing extremist propaganda and perceived it as more persuasive. We discuss the results concerning the implications for evolutionary media psychology and the transmission of parochial altruism in propaganda videos.

## Introduction

Extremist propaganda videos have become a frequent part of the contemporary online landscape. In Germany in particular, right-wing extremists such as the “Oldschool Society” and Islamic extremists such as the “Islamic State” use YouTube videos to target recipients who share their nationality or religion in order to gain new followers (Bayrisches Staatsministerium des Inneren für Bau und Verkehr, 2014). Via these videos, the propagandists try to convince the recipients that their nation (respectively, religion) is menaced by extinction (Godall, 2010; Halverson et al., 2011; Kruglanski et al., 2013) due to the “the system” or “the West” and that they have to join the propagandist’s fight and be willing to risk life and limb to preserve their group.

The so propagated behaviors of supporting one’s in-group members, even at the cost of one’s own resources (*altruism*), and of aggressively fighting out-groups (*parochialism*) has gained substantial attention in recent years from evolutionary psychological research under the concept of *parochial altruism* (Choi and Bowles, 2007; Bowles, 2008). Evidence gathered in different research areas has demonstrated that parochial aggression toward out-groups and altruistic cooperation with in-group members are deeply interwoven (Bornstein and Erev, 1994; Rusch, 2014), whereby “people go to war” (Böhm et al., 2015) to defend their fellows. It is on this defense of those sharing one’s national or religious identity against the “wicked enemy” that extremist propagators capitalize (Lasswell, 1927). Yet their open call for parochial aggression stands in sharp contrast to contemporary egalitarian norms (Pettigrew, 1995). And, of course, neither the majority of Germans nor the majority of Muslims shares the extremist propagators’ attitudes (Decker et al., 2012; Frindte et al., 2012). Prior research demonstrated propaganda to be evaluated very negatively and recipients to deny the effects of propaganda (Arendt, 2015). Moreover, in contrast to the propagators’ aims, propaganda was evaluated even more negatively when it was directed to the recipients’ national or religious in-group (versus to another audience; Rieger et al., 2013). Nevertheless, single videos *can* raise interest and become viral (Glaser, 2013), and propaganda *has been* discussed as persuading individuals from radical ideologies (Dilanian and Bennett, 2013). Although interest in propaganda does not necessarily lead to radicalization, it is a necessary precondition for further exposure to such messages (McCauley and Moskalenko, 2008; Wilner and Dubouloz, 2009) and an initial step in a potential persuasion processes (Lewis, 1903).

In the current paper, we examined the effects of extremist propaganda from an evolutionary media psychological perspective. We built upon the assumption that media allows the recipient to simulate experiences relevant to his or her level of evolutionary fitness (Tooby and Cosmides, 2001) without “risking life and limb in the real world” (Schwab and Schwender, 2010, p. 31). Media features are recognizable as “design features of an evolved system whose biological function is learning” (Vorderer, 2006, p. 14). Following Schwab (2010) media pique interest when evolutionary problems, such as the threat by predators (Tooby and Cosmides, 2001) or hostile intergroup conflicts are displayed. Thus, the conditions under which parochial altruism increases—namely, the perception of threat and vulnerability (De Dreu et al., 2010; Rusch, 2014; Böhm et al., 2015)—most plausibly also increase the interest in parochial propaganda. Note that we do not suggest that recipients are “entertained” by propaganda such as by entertaining media (Ohler and Nieding, 2006). Instead, we assume that “media events are produced by people for people, they are geared to human needs” (Schwab and Schwender, 2010, p. 21). A larger effectiveness of propaganda thus should be mirrored in a higher perceived persuasiveness of these videos.

### Parochial Altruism and the Role of Threat

From a Darwinian perspective human social behavior has developed throughout phylogenies via the adaptation to natural conditions and in order to increase the individual’s fitness and maximize its reproductive success (West et al., 2011). Acts of altruism (Hamilton, 1964a,b; Zahavi, 1995) and parochial aggression toward out-groups (Choi and Bowles, 2007) are both puzzling, as they can impair individuals’ fitness by reducing resources and hampering lucrative trading. Theories that take only an individual-level perspective on altruistic (or aggressive) behavior such as *kin selection* (Hamilton, 1964b; Riolo et al., 2001) or *reciprocal altruism* (Trivers, 1971; Axelrod and Hamilton, 1981) have failed to explain altruism to non-kin, such as the behavior called for by extremist propaganda, when reciprocation is unlikely.

Recent theories on parochial altruism are more promising for explaining, for instance, self-sacrifices in the name of one’s religion (Ginges and Atran, 2009; Ginges et al., 2009) or nation (De Dreu et al., 2014). In contrast to prior theories, the concept of parochial altruism considers both the intragroup and the intergroup level of behavior (Arrow, 2007). Humans are social animals, and group membership increases their odds for survival throughout phylogenies far beyond what would have been possible for a single individual (De Dreu et al., 2014). Thus, humans’ self-interest evolved mitigated by their group membership (Brewer and Carporael, 2006). On the intragroup level, altruistic individuals have a lower direct fitness (because altruism is costly). On the intergroup level, however, altruistic individuals increase the fitness of the group by investing more in that group and therewith increase the individuals’ indirect fitness (Arrow, 2007). Choi and Bowles (2007) provided evidence for this assumption by simulating groups of agents (tolerant versus parochial, altruistic versus selfish) that interacted with each other over thousands of generations under conditions likely to represent human interactions in early times of humankind. Violent conflicts in this simulation were likely when parochialists formed the majority of at least one group in that interaction. Furthermore, only parochial altruists (“warriors”) actively engaged in intergroup fighting (non-altruists would not be willing to do the fighting themselves, and tolerant others would prefer to peacefully interact with the out-group). The results demonstrated that groups with more parochial altruists not only engaged in more conflicts but also tended to win these wars. The societies that emerged within this simulation were stable when parochial altruists or selfish but tolerant trades formed the majority.

Of note, the willingness to parochially aggress out-groups has been observed mainly under conditions of conflict (Bornstein et al., 2002; Halevy et al., 2008; Abbink et al., 2012) when subjects perceived themselves as vulnerable (Böhm et al., 2015), wanted to protect their in-group members (Rusch, 2014), or wanted to sanction someone who had threatened their fellows before (Bernhard et al., 2006). For instance, De Dreu et al. (2010) found subjects to preemptively strike against out-group members in an intergroup prisoner dilemma only when they feared that their in-group would lose resources due to future out-group actions. This is highly compatible with social psychological research demonstrating how threat motivates intergroup biases (Hewstone et al., 2002).

Beyond threats to concrete in-group members, symbolic threats (Stephan et al., 2000; Hewstone et al., 2002; Hogg et al., 2010) can also foster parochial altruism. In particular, research inspired by terror management theory (Greenberg et al., 1986) repeatedly demonstrated existential threats resulting from reminders of one’s own mortality (mortality salience, MS) to increase the acceptance of parochially altruistic in-group members (for a review, see Pyszczynski et al., 2008). For instance, Greenberg et al. (2001) demonstrated that, in the absence of MS, white Americans evaluated a white American who claimed to be “proud of being white” as more racist than someone who claimed to be “proud of being black.” This effect disappeared under conditions of MS. Similarly, Pyszczynski et al. (2006) found US participants to be more accepting of violent military attacks on Muslim out-group members, and Iranian students to offer a more favorable evaluation of someone expressing parochial altruistic anti-US and pro-martyrdom attitudes under conditions of MS.

With its parochially altruistic content, extremist propaganda most plausibly reaches its targeted audience only after a perceived threat has made these recipients vulnerable to the parochially aggressive narrative. We tested this assumption by conceptually replicating the study by Rieger et al. (2013) on the evaluation of right-wing extremist and Islamic extremist propaganda videos. More precisely, we compared the effects of MS versus a control topic on the evaluation of these propaganda videos in a German student sample. Rieger et al. (2013) found German students to report less interest in and persuasiveness of right-wing extremist as compared to Islamic extremist propaganda, but we predicted that, under conditions of threat, German students would report increased interest (H1) in the right-wing extremist propaganda and perceive the videos as more persuasive (H2). Moreover, increased interest should be positively associated with an increased persuasiveness ascribed to these videos (H3).

Beyond our central questions, our study also had a pair of secondary objectives. First, we expected the effects of MS to represent a general response to parochial altruistic propaganda addressing them as in-group members via their nationality. Consequently, we expected the effects to explain additional variance beyond political or ideological attitudes (e.g., authoritarianism) that have been reported previously to predict interest in extreme ideologies (Altemeyer and Hunsberger, 1992; Fuchs, 2003; Sibley and Duckitt, 2008; Rieger et al., 2013). Second, we wanted to check for gender differences. Prior research often relied on male samples for studying parochial altruism (De Dreu, 2012) or the effects of extremist propaganda (Rieger et al., 2013). Studies including both genders report mixed results. Some studies find stronger parochial aggression among males (Yuki and Yokota, 2009) and parochial aggression to be positively associated with levels of testosterone (Reimers and Diekhof, 2015). Other studies report no gender differences in the acceptance of parochial altruism (Ginges et al., 2009). Finally, Rieger et al. (2013) identified three more factors on which the evaluation of extremist propaganda varied: *shame* and *aversion* after the reception and the *one-sidedness* ascribed to the propaganda videos. They report German students to respond with increased levels of shame to right-wing extremist videos and to evaluate these videos as more one-sided than Islamic extremist videos (aversion ratings did not differ). We wanted to explore whether MS would attenuate these findings.

## Materials and Methods

We examined our predictions by presenting German students under conditions of MS (versus a control topic) with parochially altruistic extremist propaganda, addressing them as in-group members (through right-wing extremist videos targeting “the Germans”) or as out-group members (through Islamic extremist videos targeting “the Muslims”). The last factor served as a within-subjects factor.

### Sample

G*Power calculated that a sample of *N* = 92 would be necessary to prove the smallest effect size observed by Rieger et al. (2013) for interest in right-wing extremist propaganda (*r* = 0.19). A total of 114 subjects finished our study (drop out *n* = 33). To hold the group association between sender and recipient constant, we recruited only subjects who were born in Germany and did not self-identify as Muslims. Five participants did not fulfill these sampling criteria and were excluded from the analyses. The remaining *N* = 109 (all German non-Muslims, 18 male) were on average 25.17 years old (SD = 6.34). The majority of them (94.4%) were students, while the remaining participants were already in the workforce. Gender, current profession (both χ^2^ > 1), and age (*F* < 1) did not vary depending on condition. The majority of our participants self-classified as Christians (74%) or atheists (23%); three subjects reported “another” religion. Religion was equally distributed among conditions, χ_exact_^2^(3) = 3.50, *p* > 0.20. On an 11-point scale (0 = “totally unimportant,” 10 = “totally important”) subjects rated their religion as rather unimportant for them (*M* = 3.08, SD = 2.81). Only two of the participants rated religion as “totally important” to them. Relevance of religion did not vary between conditions, *F* < 1. On a 10-point scale (1 = “left-wing,” 10 = “right-wing”), subjects were rather left-wing oriented (*M* = 4.20, SD = 1.38). None of the participants was extremely right-wing oriented (Range 1–8). Political attitudes did not vary between conditions, *F* < 1.

### Procedure and Materials

Subjects were invited via German university mailing lists to participate in an online experiment about “political videos on YouTube.” We rewarded them with the opportunity to participate in a lottery for two Amazon.de vouchers, each worth 30€. At the beginning of the questionnaire, subjects confirmed that they were over 18 years old and that they had read, understood, and accepted the ethical consent form. Afterward, they answered a set of demographic (age, nationality, religious identity, political attitude) and attitudinal questionnaires. To confirm that the effects we found were not solely attributable to interpersonal differences associated with hostile intergroup attitudes, subjects filled out a measure of *authoritarianism* (Petzel et al., 1997); *violence acceptance* (Wagner et al., 2002); *anomia*, their feeling of value lost (Fuchs, 2003); and *self-esteem* (Rosenberg, 1965). Subsequently, subjects were randomly assigned to either the MS or a control condition.

#### Salience Manipulation

Participants in the MS condition (*n* = 57) answered the standard two open-ended questions used in terror management research: (1) “Please briefly describe the emotions that the thought of your own death arouses in you.” (2) “Please describe, as specifically as you can, what you think will happen to you as you physically die and once you are physically dead” (Rosenblatt et al., 1989). Participants in the control condition (*n* = 41) were given the same instructions, but the references to death were replaced with references to “failing an exam” (Monin, 2009).

#### Delay

Mortality salience affects intergroup attitudes only distally to death reminders, when the death-related thoughts are no longer in focal attention (Pyszczynski et al., 1999). Hence, to enable their distal defense subjects worked on a set of 35 raven matrices (Raven, 1998) before the next part of the experiment started.

#### Video Exposure

After the last matrix, participants watched two blocks of extremist videos in randomized order. Each block comprised three videos, either from right-wing extremists (total duration 07:58 min.) or from Islamic extremists (total duration 07:36 min.). We held the formats of the videos constant between the ideologies. We selected the videos from the database by Rieger et al. (2013). The videos in this database did not show explicit depictions of physical violence (such as beheadings) and were approved by the ethics committee of the German Federal Crime Police Office prior to data collection in their studies. Subjects in our study saw one *talking head lifestyle activist* video, one *movie clip* video, and one *extreme clip* video (see Supplementary Table S1, for a summary of their content).

#### Dependent Measures

Participants rated each video on the five scales that Rieger et al. (2013) introduced. The scales measured the participants’ *interest* (e.g., “The video was interesting”) in the videos, the videos’ *perceived persuasiveness* (e.g., “After the video, I can understand the perspective of its producers better”), and the level of *shame* (“During the reception I felt shame”) triggered by the video. Furthermore, we also measured subjects’ level of *aversion* during the reception (e.g., “During the reception I felt disgust”) and the *one-sidedness* ascribed to the video (e.g., “The video was sensational”), to ensure conditions similar to those in the studies by Rieger et al. (2013). Each of the 14 total items was evaluated on a four-point scale (1 = “totally not,” 4 = “totally”).

#### Check for Suspicion

Finally, subjects were checked for suspicion and watched a video debriefing (05:06 min) by the first author, supplemented by a written debriefing and the author’s contact details.

## Results

### Data Aggregation

To ensure that participants had watched the videos, we subtracted the actual length of the video from the time subjects spent on the corresponding page. Subjects who did not watch the whole video received a negative difference; subjects who proceeded with the video after its end received a positive value (due to the response latency between the end of the video and the key pressure). To control for outliers, these scores were then *z*-standardized. Subjects with a negative value or with *z* > 3 were treated as missing values for the respective video evaluation. Following the procedure by Rieger et al. (2013), we then computed mean scores for each of the dependent variables per ideology, resulting in one value per scale for both the right-wing extremist and the Islamic extremist videos. For the perceived persuasiveness ratings, the internal consistency for the aggregated right-wing extremist as well as the Islamic extremist videos was slightly questionable, both α = 0.65. All other scales α > 0.70.

### Preliminary Analyses

We first examined the association of the included variables with each other via Pearson correlations. Political attitude, anomia, and violence acceptance were not significantly associated with the dependent variables, all *r* < 0.20, hence these variables were excluded from the analyses thereafter (Field, 2013). Preliminary analyses of variance (ANOVAs) using the participants’ characteristics as dependent variables revealed that subjects in the MS as compared to the control condition reported lower levels of self-esteem, *F*(1,107) = 8.24, *p* < 0.05, *r* = 0.27. Consequently, the assumptions of analysis of covariance were not met (Field, 2013). Subjects did not differ regarding their level of authoritarianism, *F* < 0.1.

Based on these findings, we analyzed the video evaluation via separate hierarchical regression analyses (see Weise et al., 2008 for a similar approach). Block 1 contained all variables measuring interindividual differences (*z*-standardized) following the recommendation that predictors based on prior research should be entered first to partialize their effects out before the predictive value of the experimental manipulation is assessed (Field, 2013). Condition (dummy coded, 0 = control, 1 = MS) was entered in Block 2. To assess potential moderations, the two-way interactions between condition, authoritarianism, and self-esteem were entered in Block 3. Parameter estimates were based on 1000 bootstrap samples. The results for the simplest model including only condition as a predictor are provided in the Supplementary Table S2.

### Hypotheses Testing

Regarding interest in the right-wing extremist videos, interindividual differences (Block 1) and their interactions with the condition (Block 3) failed to explain variance. As predicted in H1, condition (Block 2) significantly predicted interest, *F*_change_(1,96) = 4.27, *p* < 0.05. Subjects under conditions of MS reported more interest in the right-wing extremist propaganda than did subjects in the control condition. None of the models explained the interest in the Islamic extremist videos, all *p* ≥ 0.20.

Hypothesis 2 predicted that subjects under conditions of MS would perceive the in-group propaganda as being more persuasive. Block 1 (containing the interindividual differences) reached significance, *F*_change_(4,94) = 3.17, *p* < 0.05. Higher levels of authoritarianism predicted higher perceived persuasiveness of the right-wing extremist videos. What is more relevant, Block 2 also reached significance, *F*_change_(1,93) = 6.92, *p* = 0.01. Subjects in the MS condition perceived the extremist in-group messages to be more persuasive than did control subjects. The effect of authoritarianism remained stable. Block 3 failed to explain further variance (see **Table 1**). None of the models explained the perceived persuasiveness of the Islamic extremist videos, all *p* ≥ 0.20.

**Table 1:** Regression analyses for interest and persuasiveness ratings after the right-wing extremist videos.

		Block 1					Block 2					Block 3				
		*b*	LL	UL	SE	β	*b*	LL	UL	SE	β	*b*	LL	UL	SE	β
**Interest**
	Constant	1.68	1.43	1.98	0.14		1.53	1.28	1.82	0.14		1.53	1.26	1.82	0.14
	Age	0.00	–0.11	0.13	0.06	0.00	0.00	–0.12	0.12	0.06	0.00	–0.01	–0.13	0.12	0.06	–0.01
	Gender	0.02	–0.31	0.30	0.16	0.01	0.02	–0.29	0.30	0.15	0.01	0.01	–0.29	0.30	0.16	0.01
	Authoritarianism	0.15	–0.08	0.35	0.11	0.15	0.16	–0.08	0.36	0.11	0.16	0.13	–0.23	0.54	0.19	0.12
	Self-Esteem	–0.04	–0.20	0.15	0.09	–0.05	0.00	–0.15	0.18	0.09	0.00	0.07	–0.22	0.32	0.13	0.08
	MS versus Control						**0.25**	**0.004**	**0.46**	**0.12**	**0.21^∗^**	**0.26**	**–0.02**	**0.47**	**0.12**	**0.22^∗^**
	MS × Authoritarianism											0.05	–0.47	0.52	0.24	0.04
	MS × Self-Esteem											–0.10	–0.40	0.30	0.18	–0.09
			*R*^2^ = 0.02					*R*_change_^2^ = 0.04^∗^					*R*_change_^2^ = 0.00				
**Persuasiveness**
	Constant	1.46	1.30	1.615	0.08		1.33	1.15	1.49	0.09		1.34	1.16	1.52	0.09
	Age	–0.02	–0.13	0.075	0.05	–0.04	–0.02	--0.13	0.07	0.05	–0.05	–0.02	–0.14	0.07	0.05	–0.05
	Gender	0.09	–0.10	0.281	0.10	0.07	0.09	–0.08	0.28	0.09	0.07	0.09	–0.08	0.30	0.10	0.07
	Authoritarianism	**0.24**	**0.06**	**0.410**	**0.09**	**0.31^∗∗^**	**0.25**	**0.07**	**0.41**	**0.08**	**0.32^∗∗^**	0.20	–0.06	0.44	0.13	0.26
	Self-Esteem	–0.07	–0.21	0.066	0.07	–0.11	–0.03	–0.16	0.09	0.06	–0.05	–0.06	–0.34	0.16	0.13	–0.09
	MS versus Control						**0.23**	**0.07**	**0.38**	**0.08**	**0.26^∗∗^**	**0.22**	**0.05**	**0.38**	**0.08**	**0.25^∗∗^**
	MS × Authoritarianism											0.07	–0.27	0.42	0.17	0.07
	MS × Self-Esteem											0.05	–0.22	0.37	0.15	0.06
		*R*^2^ = 0.12^∗^					*R*_change_^2^ = 0.06^∗^					*R*_change_^2^ = 0.00				

*^∗∗^*p* ≤ 0.01 (two-tailed), ^∗^*p* ≤ 0.05 (two-tailed). Significant predictors are marked in bold face. Confidence intervals and SE are based on 1000 bootstrap samples. MS = mortality salience.*

In line with the expectation formulated in H3, correlational analyses showed that interest and perceived persuasiveness for the right-wing extremist videos were strongly associated in the MS condition = 0.67, *p* < 0.001, and had a lower but still significant association in the control condition, *r* = 0.30, *p* < 0.05.

### Additional Analyses

Following the procedure by Rieger et al. (2013), our study also included measures of shame, aversion, and one-sidedness. MS had no effects, however, on either reported aversion or one-sidedness ascribed to the videos. Regarding shame after the right-wing extremist videos, only Block 2 (condition) reached marginal significance, *R*_change_^2^ = 0.04, *F*_change_(1,93) = 3.70, *p* < 0.06. Subjects in the MS condition reported more shame after the right-wing extremist messages than did subjects in the control condition (*b* = 0.22, SE = 0.10, β = 0.20, CI [–0.33, 0.05]; see Supplementary Table S3). All other models failed to reach significance, all *p* > 0.20. Shame reported after the Islamic extremist videos, in contrast, was significantly predicted by Block 1, *R*^2^ = 0.13, *F*_change_(4,93) = 3.39, *p* = 0.01. Higher self-esteem predicted lower levels of shame (*b* = –0.30, SE = 0.08, β = –0.36, CI [–0.45, –0.13]). All other models failed to reach significance, all *p* ≥ 0.20. Pearson correlations showed that shame had a moderate association with interest, *r* = 0.33, *p* < 0.05, and perceived persuasiveness, *r* = 0.43, *p* < 0.001, in the MS condition but not in the control condition (all *p* > 0.10).

**FIGURE 1:**
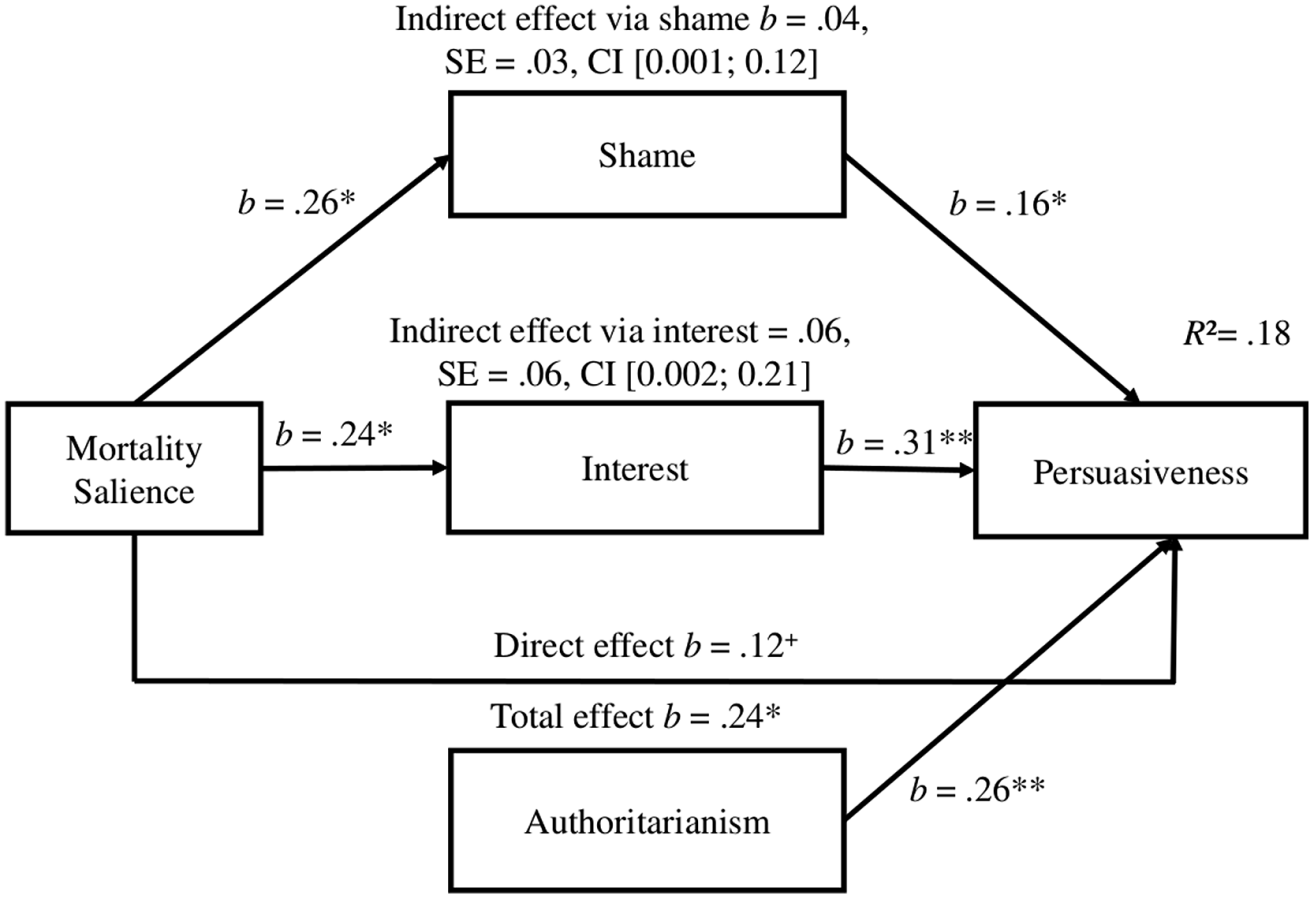
**The effect of mortality salience on the perceived persuasiveness of right-wing extremist videos mediated by shame and interest.** ^∗∗^*p* > 0.01, ^∗^*p* > 0.05, ^+^*p* > 0.10. Parameter estimates are based on 1000 bootstrap samples.

The results so far suggest that interest and shame might increase the perceived persuasiveness of the videos. We explored this idea via a mediation analysis using the PROCESS macro by Hayes (2012). We entered condition as a predictor variable (0 = control, 1 = MS), interest and shame as mediators, and authoritarianism as covariate (*z*-standardized). The results showed that the effect of MS on the perceived persuasiveness of the right-wing propaganda was significantly mediated by interest, *ab* = 0.08, SE = 0.06, CI [0.002, 0.21], but also by shame, *ab* = 0.04, SE = 0.03, CI [0.005, 0.12]. The contrast between these two failed to reach significance, C_1_ = 0.03, SE = 0.07, CI [–0.07, 0.18]. Meanwhile, the total effect of MS on perceived persuasiveness was significant; the direct effect when both mediators were included was only marginally significant (see **Figure 1**).

## Discussion

Right-wing extremists and Islamic extremists propagate parochial altruism to recipients sharing their national or religious identity via Internet videos. Evolutionary media psychology posits that the response to media content works on evolutionary mechanics, whereby media content displaying evolutionary relevant problems (such as parochial altruism in hostile intergroup interactions) creates larger interest (Tooby and Cosmides, 2001; Schwab and Schwender, 2010). Drawing upon research finding that threats increase parochial altruistic behavior in humans, we predicted that existential threats—namely, reminders of one’s mortality—would increase the interest in and the perceived persuasiveness of extremist propaganda. Overall, the results confirmed our expectations. German students reported an increased interest in right-wing extremist videos under conditions of MS (Hypothesis 1) and ascribed these videos a larger persuasiveness (Hypothesis 2). Interest and perceived persuasiveness were positively associated (Hypothesis 3). Moreover, additional mediation analyses demonstrated that interest partially mediated the association between MS and perceived persuasiveness. The effects of MS emerged after interpersonal differences in factors such as authoritarianism, self-esteem, or gender were partialized out, underlining the assumption that the response to parochial altruistic in-group members represents a general mechanism. Overall, our findings confirm prior research on parochial aggression (De Dreu et al., 2010; Rusch, 2013; Böhm et al., 2015) and the acceptance of parochially aggressive in-group members (Pyszczynski et al., 2006; Kugler and Cooper, 2010) after MS, and extended these findings to the area of media psychology. Further, MS did not affect the response toward the Islamic extremist videos underlining the crucial role of being addressed as in-group member (and not, for instance, the interest in extremist messages *per se* (Hogg, 2014).

Beyond our predictions, we also found MS to increase participants’ levels of shame after exposure to propaganda videos addressing the recipient as an in-group member. Shame further partially mediated the effects of MS on the perceived persuasiveness of the videos. Albeit prior evidence on the relationship between shame and hostile intergroup attitudes is somewhat mixed (Brown et al., 2008; Piff et al., 2012), our results confirm studies showing that shame can motivate parochial aggression (Tangney et al., 1992; Lickel et al., 2005). Nevertheless, the bootstrapped confidence intervals encompassed zero, so our findings should be interpreted cautiously and future studies exploring the role of shame in more detail are necessary.

Overall, our results are meaningful on both a theoretical and an applied level. On a theoretical level, they transfer the perspective of parochial altruism to media psychology. In so doing, they extend earlier theories by showing that not only entertainment (Ohler and Nieding, 2006; Schwab, 2007) but also the response to hostile media evolved from evolutionary adaptive processes. In light of our results, propaganda might be the medium that makes “people go to war” (Böhm et al., 2015). Moreover, our results fit current communication research demonstrating that media satisfy basic human needs (Bartsch and Schneider, 2014; Roth et al., 2014). Our study suggests that incorporating evolved needs into such theory building might enhance our understanding of media consumption and effects.

Moreover, our findings add to the growing evidence that media serve an anxiety-buffering function (Rieger et al., 2015). Notably, even under conditions of threat, subjects did not react enthusiastically to the propaganda messages; the effects remained small. Yet our finding matches prior studies showing that individuals (in experimental research) overall are not very hostile toward out-group members at all; instead, parochial aggression manifested, for instance, in the refusal to help out-group members (Weisel and Böhm, 2015). Luckily, the exclusive reliance on parochial altruistic behaviors is relatively seldom (De Dreu et al., 2015).

Nevertheless, not all individuals have to serve as “warriors” in intergroup conflicts; accepting them as the dominant group also allows them to foster violent intergroup encounters (Choi and Bowles, 2007). From a media psychological perspective, allowing extremists to voice their opinion might, via spiral process, create the illusion that they already form the majority and therefore reduce anti-extremist voices (Glynn et al., 1997).

On an applied level, the wide distribution of extremist propaganda makes our effects although they are small interesting for practitioners. For instance, our effects were irrespective of gender, suggesting that the current susceptibility of young women listening to the propaganda of Islamic extremists reported by mass media (Wahba and Simon, 2014) might also work on evolved mechanics. Research focusing on gender differences in the context of parochial altruism and extremism could provide meaningful insights here.

In addition, understanding the parochial altruistic mechanisms of propaganda effects could help to attenuate the influence of such videos. For instance, the salience of a certain social category (such as resulting by being addressed as “German” in a right-wing extremist video) is a fluent process and depends on the (potential) cooperation between in-group members. Albeit, we observed MS to increases the interest in extremist messages even among recipients with moderate political attitudes, as long as the videos capitalized on the shared social category, such categorizations are not stable. Kurzban et al. (2001) found that even dominant cues such as ethnicity (Cosmides et al., 2003; Xiaojing et al., 2009) are attenuated by making other group memberships salient. Consequently, distributing so-called *counter-narratives* (Ashour, 2010) capitalizing on *shared* group identities (e.g., being human) could enhance altruism toward others beyond one’s national group (Pyszczynski et al., 2008).

Further, in the control group no interest in the parochial in-group propaganda was observed suggesting that removing threat could foster peace (Böhm et al., 2015). But also under conditions of threat, research demonstrating the effects of MS to be sensitive to salient norms (Jonas et al., 2008; Schindler et al., 2013), suggest that making norms of tolerance salient, for instance via counter-narrative media, could reduce the interest in parochial propaganda (Pyszczynski et al., 2008).

### Limitations

Some limitations of the current study have to be noted. First, we used a typical student sample. Rieger et al. (2013) found opposed reactions by students and apprentices, therefore a replication of our study in a non-academic sample would be desirable. Prior research identified students as particularly non-susceptible to in-group extremist propaganda; thus, the effects of MS we observed in this sample are particularly meaningful. Accordingly, replicating our study in a Muslim sample who are addressed as “in-group members” in Islamic-extremist videos would extend the generalizability of our findings. Noteworthy, parochial altruism as an evolutionary adaptive response should not depend on the cultural background of the recipients per se. Accordingly, MS has already been demonstrated to increase the acceptance of parochial aggressive in-group members among Iranian students (Pyszczynski et al., 2006). Further, we did not measure participants’ subjective identification with nationality. Research has found that people highly committed to their group are more likely to accept parochial altruism (here: religious martydom; Ginges et al., 2010), so including such measures in future studies seems desirable. Nonetheless, parochial altruism has been observed in both minimal and real groups, suggesting that subjective identification is not enough to explain the response to parochially altruistic in-group members.

Regarding our design, it has to be pointed out that subjects participated in an anonymous online questionnaire. Although this format is highly compatible with real-life exposure to extremist Internet propaganda, we cannot dismiss the possibility that subjects might display different reactions oﬄine. For instance, social identity de-individuation theory (Postmes et al., 1998) found subjects in anonymous online interactions to be even more prone to behaving according to situationally salient group memberships. Consequently, the evaluation of in- but also out-group extremist propaganda might vary depending on whether a person watches such material alone online or together with others.

Concerning our materials, it has to be pointed out that we did not find effects of MS on the response to out-group propaganda. At first sight, this contradicts studies showing harsher punishment of out-group terrorists under conditions of MS (Kugler and Cooper, 2010) or harsher punishments of out-group than of in-group perpetrators due to parochial altruistic motivations (Bernhard et al., 2006). However, we focused on positive responses to parochial aggressive propaganda and did not analyze the response to media narratives displaying the *punishment* of out-groups. Our findings imply that such narratives (e.g., killing out-group terrorists in *Homeland*) would also raise interest and be perceived as more persuasive (see Slater and Rouner, 2002, for the concept of narrative persuasion) due to parochially altruistic motivations. Furthermore, it should be noted that extremist propaganda itself can be regarded as threatening, and studies have shown that terrorism itself can induce death anxieties and trigger MS effects (Fischer et al., 2007; Das et al., 2009). Consequently, our participants might have perceived the Islamic extremist videos as more threatening than the right-wing extremist videos. Yet Rieger et al. (2013) found no significant differences in physiological arousal during the reception of the Islamic versus right-wing extremist videos, making this explanation implausible. Further, our selection differentiated between extremist videos offering the recipient to join their cause versus not. MS has been found to increase affiliation (Wisman and Koole, 2003). Consequently, examining the role of affiliation motives in this context in future studies is necessary to compare the turning toward different groups after MS.

As regards to our dependent variables, we focused on the interest in and the perceived persuasiveness of the videos. Although we based our dependent variables on prior research, these variables have to be interpreted cautiously. Interest is only an initial step in a potential process that might increase parochial attitudes and should not be interpreted as a direct measure of parochial altruism. Interest reflects the motivation to get more information about a certain topic (Schwab and Schwender, 2010) and could also reflect an increased desire to restore a sense of control in face of a threat induction (Fritsche et al., 2008; Jonas et al., 2014). However, if the desire to know more about a potential threat instead of the interest in parochial altruistic content would underlie our pattern, we would also find larger interest in the Islamic extremist videos, as Islamic extremism is perceived as particularly threatening (Frindte and Haußecker, 2010).

In that line it has to be noted that the reliability of our perceived persuasiveness measure aggregated for the video blocks was slightly below α > 0.70. Although previous research obtained higher reliability scores for this scales (Rieger et al., 2013), and the reliability was good, α > 80, when all items per video instead of the aggregated scales per Block were considered, future studies should include additional measures of persuasiveness. Overall, future research is necessary to bridge our findings on the perceived persuasiveness of extremist propaganda with research on liking of parochial altruist persons (Greenberg et al., 2001; Pyszczynski et al., 2006). Our scale measured increased sympathy with the propagators, hence, our findings should also be reflected in greater liking of extremist propagators (Decety and Chaminade, 2003). Relatedly, we did not measure attitudes toward the propagated message directly. Yet, Igartua et al. (2003) found that convincingness of a persuasive video was associated with agreement to these videos’ messages and we have initial evidence from an unpublished Bachelor thesis that our perceived persuasiveness scale is associated with the agreement to right-wing statements. Kasztelan (unpublished bachelor thesis) found that the perceived persuasiveness of right-wing videos correlated at *r* > 0.56, *p* > 0.001 with the agreement to statements in a propaganda video such as “our folk impoverishes everyday more and more meanwhile others live in clover” or “Only a folk without identity becomes an easy victim of the capital.” Finally, studying to what extent MS affects hormones that control parochial altruism such as oxytocin (De Dreu et al., 2010, 2011) or testosterone (Reimers and Diekhof, 2015) would provide meaningful insights into the biological mechanisms underlying our observations.

## Conclusion

Overall, our study provides initial evidence that the interest in and perceived persuasiveness of extremist propaganda works according to a parochially altruistic mechanics. Existential threats affected the response to extremist propaganda capitalizing on the recipient’s national identity but left the response to comparable videos addressing them as out-group members unaffected. Our study thus provided evidence for meaningful insights resulting from an evolutionary perspective on media psychology and propaganda research. We hope that future studies will address the questions that can be drawn from our results.

## Conflict of Interest Statement

The authors declare that the research was conducted in the absence of any commercial or financial relationships that could be construed as a potential conflict of interest.
