# Supplementary material for: Dying the right-way? Interest in and perceived persuasiveness of parochial extremist propaganda increases after mortality salience
Source: Front Psychol. 2015 Aug 14;6:1222. doi: 10.3389/fpsyg.2015.01222 (PMC4536404; doi:10.3389/fpsyg.2015.01222)
Supplement: Supplementary file 1 [file Table_1.DOCX]

***Supplementary Material***

**Dying the right-way? Interest in and perceived persuasiveness of parochial extremist propaganda increases after mortality salience**

**Table 1. Short summaries of the video stimuli.**

|  | **Video** | **Duration** | **Summary** |
| --- | --- | --- | --- |
| Right-wing extremist | Talking head lifestyle activist | 0:02:04 | The video shows three young autonomous nationalists that walk through a German city. Iterating one of them talks directly to the camera. The sequence starts with the call "German open your eyes, you are in War. The system fosters interracial marriages because they do not give birth to German children [...]", addresses drug abusion (“weakening the Germans”), the “hostile” media and ends with the claim "national resistance, join us! After our victory, never ever war", shouted by a demonstration of autonomous nationalists dressed in Black. |
|  | Movie clip | 0:02:35 | The video addresses the bombing of the German city Siegen during World War II. It shows black and white drawings of the skyline of Siegen and falling bombs, combined with historic pictures of crying women, dead children and ruins. The pictures are underlined with dramatic music and accompanied with written statements about the victims of this bombing. The videos ends with the picture of two bombs labelled as "democracy”, and “liberty”, together with the headline "we did not forget how you freed us". |
|  | Extreme clip | 0:03:19 | The video addresses sexual abuse by combining mass media pictures of children that became victims with a song about the raping of a little girl. It ends with the demand of "harsher punishments for pedophiles" and pictures of a demonstration of autonomous nationalists accompanied with parts of the "pirates of the Caribbean" soundtrack. |
| Islamic extremist | Talking head lifestyle activist | 0:02:46 | The video shows a young German Jihadist who talks directly to the camera. He asks “how can I remain seated after I read the letter of Fathima from Iraq, her letter from Abu Grahib where she writes that she has been raped nine times on a single day by these perverted animals […]. Mother, a Muslima!" he talks about the incidents in Iraq and the cooperation between some countries in the Middle east and the USA. The scene ends with a claim that "they tank their airplanes that bomb our brothers in Iraq over there. Mother how shall I remain seated?" |
|  | Movie clip | 0:02:48 | The video shows pictures from the bombing of Iraq combined with pictures of parents carrying their dead children and crying women. The pictures are accompanied by dramatic music. The subtitles lament about the suffering of the Muslim community and accuse the USA of being liars and aggressors. The video ends with demonstrations of the organization behind the video around the world combined with the "pirates of the Caribbean theme". |
|  | Extreme clip | 0:02:02 | The video shows the preparation of a suicide attack in the middle east. The car with the bomb is shown, accompanied with a religious song (nasheed) about the paradise awaiting the perpetrator. The video ends with a long camera shot to a city at the horizon where an explosion takes place. |
