# Supplementary material for: Dying the right-way? Interest in and perceived persuasiveness of parochial extremist propaganda increases after mortality salience
Source: Front Psychol. 2015 Aug 14;6:1222. doi: 10.3389/fpsyg.2015.01222 (PMC4536404; doi:10.3389/fpsyg.2015.01222)
Supplement: Supplementary file 2 [file Table_2.DOCX]

***Supplementary Material***

**Dying the right-way? Interest in and perceived persuasiveness of parochial extremist propaganda increases after mortality salience**

**Table 2. Regression analyses including only condition as predictor for all dependent variables.**

|  |  | Right-wing extremist videos | | | | |  | Islamic extremist videos | | | | |
| --- | --- | --- | --- | --- | --- | --- | --- | --- | --- | --- | --- | --- |
|  |  | b | LL | UL | SE | β |  | b | LL | UL | SE | β |
| Interest | Constant | 1.55 | 1.39 | 1.73 | 0.09 |  |  | 1.81 | 1.64 | 1.99 | 0.09 |  |
|  | MS versus Control | **0.24** | **0.01** | **0.46** | **0.11** | **0.20*** |  | 0.00 | -0.23 | 0.23 | 0.12 | 0.00 |
|  |  | *R*² = .04* | | | | |  | *R*² = .00 | | | | |
| Persuasiveness | Constant | 1.40 | 1.29 | 1.50 | 0.06 |  |  | 1.69 | 1.53 | 1.85 | 0.08 |  |
|  | MS versus Control | **0.22** | **0.07** | **0.37** | **0.08** | **0.25*** |  | 0.08 | -0.11 | 0.25 | 0.09 | 0.09 |
|  |  | *R*² = .06* | | | | |  | *R*² = .01 | | | | |
| Shame | Constant | 1.60 | 1.45 | 1.76 | 0.08 |  |  | 1.66 | 1.50 | 1.84 | 0.09 |  |
|  | MS versus Control | **0.27** | **0.04** | **0.49** | **0.11** | **0.24*** |  | 0.17 | -0.05 | 0.39 | 0.11 | 0.15 |
|  |  | *R*² = .06* | | | | |  | *R*² = .02 | | | | |
| Aversion | Constant | 2.88 | 2.68 | 3.09 | 0.11 |  |  | 2.64 | 2.44 | 2.85 | 0.10 |  |
|  | MS versus Control | -0.03 | -0.32 | 0.24 | 0.14 | -0.02 |  | -0.02 | -0.30 | 0.23 | 0.13 | -0.02 |
|  |  | *R*² = .00 | | | | |  | *R*² = .00 | | | | |
| One-sidedness | Constant | 2.97 | 2.84 | 3.10 | 0.07 |  |  | 2.65 | 2.47 | 2.84 | 0.10 |  |
|  | MS versus Control | 0.15 | -0.05 | 0.33 | 0.09 | 0.14 |  | 0.10 | -0.14 | 0.35 | 0.12 | 0.09 |
|  |  | *R*² = .02 | | | | |  | *R*² = .01 | | | | |
| *Notes.* * *p* ≤.05. Significant predictors are marked in bold face. Confidence intervals and standard errors are based on 1000 bootstrapp samples | | | | | | | | | | | | |
