# Supplementary material for: Dying the right-way? Interest in and perceived persuasiveness of parochial extremist propaganda increases after mortality salience
Source: Front Psychol. 2015 Aug 14;6:1222. doi: 10.3389/fpsyg.2015.01222 (PMC4536404; doi:10.3389/fpsyg.2015.01222)
Supplement: Supplementary file 3 [file Table_3.DOCX]

***Supplementary Material***

**Dying the right-way? Interest in and perceived persuasiveness of parochial extremist propaganda increases after mortality salience**

**Table 3. Regression analyses for shame ratings after the right-wing extremist videos**

|  |  | Block 1 | | | | |  | Block 2 | | | | |  | Block 3 | | | | |
| --- | --- | --- | --- | --- | --- | --- | --- | --- | --- | --- | --- | --- | --- | --- | --- | --- | --- | --- |
|  |  | b | LL | UL | SE | β |  | b | LL | UL | SE | β |  | b | LL | UL | SE | β |
| Shame | Constant | 1.60 | 1.32 | 1.88 | 0.14 |  |  | 1.47 | 1.14 | 1.78 | 0.16 |  |  | 1.47 | 1.13 | 1.79 | 0.17 |  |
|  | Age | -0.03 | -0.16 | 0.08 | 0.06 | -0.05 |  | -0.03 | -0.17 | 0.07 | 0.06 | -0.06 |  | -0.03 | -0.18 | 0.07 | 0.06 | -0.05 |
|  | Gender | 0.18 | -0.13 | 0.48 | 0.15 | 0.11 |  | 0.18 | -0.13 | 0.51 | 0.16 | 0.11 |  | 0.20 | -0.14 | 0.51 | 0.16 | 0.12 |
|  | Authoritarianism | 0.01 | -0.17 | 0.26 | 0.11 | 0.01 |  | 0.01 | -0.17 | 0.28 | 0.12 | 0.01 |  | 0.06 | -0.28 | 0.37 | 0.17 | 0.06 |
|  | Self-esteem | **-0.17** | **-0.33** | **0.05** | **0.10** | **-0.20^+^** |  | -0.13 | -0.30 | 0.08 | 0.10 | -0.15 |  | -0.16 | -0.55 | 0.24 | 0.20 | -0.19 |
|  | MS versus Control | |  |  |  |  |  | **0.22** | **-0.01** | **0.45** | **0.12** | **0.20^+^** |  | **0.22** | **-0.04** | **0.46** | **0.12** | **0.19^+^** |
|  | MS × Autoritarianism | | |  |  |  |  |  |  |  |  |  |  | -0.07 | -0.45 | 0.44 | 0.23 | -0.05 |
|  | MS × Self-esteem | |  |  |  |  |  |  |  |  |  |  |  | 0.04 | -0.39 | 0.51 | 0.23 | 0.04 |
|  |  | *R*² = .06 | | | | |  | R_change_² = .04^+^ | | | | |  | R_change_² = .00 | | | | |
| *Notes*. ^+^ *p* ≤.07 (two-tailed). Significant predictors are marked in bold face. Confidence intervals and standard errors are based on 1000 bootstrapp samples. | | | | | | | | | | | | | | | | | | |
